# The Effect and Mechanism of TLR9/KLF4 in FFA-Induced Adipocyte Inflammation

**DOI:** 10.1155/2018/6313484

**Published:** 2018-12-18

**Authors:** Cuizhe Wang, Meixiu Zhang, Jinxiu Wu, Wei Li, Xiaodan Ha, Yajuan Gu, Bo Han, Jianxin Xie, Jun Zhang

**Affiliations:** ^1^Shihezi University, School of Medicine, Shihezi, Xinjiang 832000, China; ^2^Shihezi University School of Medicine in the First Affiliated Hospital Clinical Laboratory, Shihezi, Xinjiang 832000, China; ^3^Shihezi University School of Medicine in the First Affiliated Hospital Department of Obstetrics and Gynecology, Shihezi, Xinjiang 832000, China; ^4^Shihezi University, School of Pharmacy, Shihezi, Xinjiang 832000, China

## Abstract

**Objective:**

Current research has reported that obesity is a chronic inflammatory state, which is closely related with excessive accumulation of free fatty acid, while the specific mechanism that high level of FFA causes inflammation is not very clear. Thus, our research intended to observe the high FFA effects on TLR9/KLF4 expression and the downstream inflammatory factors, to explore the mechanism of inflammatory response suppressed by TLR9/KLF4.

**Methods:**

qRT-PCR and Western blot were used to detect the mRNA and protein expression levels of TLR9, KLF4, and key inflammation-related factors. ELISA was used to detect the release level of inflammatory cytokines. The triglyceride (TG) and glucose (GLU) testing cassettes were used to detect the TG and GLU levels in culture medium.

**Results:**

In the omental tissue of obese individuals (OB), we found that TLR9, KLF4, mRNA, and the protein expression levels were lower than those of the normal weight control (NC) group. Similarly, in the omental tissue of high-fat diet (HFD) rats, we found that the mRNA expression levels of TLR9 and KLF4 were lower than those of the normal diet control group. In mature adipocytes, we found that KLF4 played an important anti-inflammatory role; moreover, PA can promote the development of inflammation by inhibiting KLF4 expression; TLR9 has a positive regulation function on KLF4 expression, but unrelated to PA.

**Conclusions:**

TLR9/KLF4 is involved in regulating FFA-induced adipocyte inflammation.

## 1. Introduction

Obesity refers to the excessive accumulation or abnormal distribution of fat, usually associated with weight gain, which has become a worldwide health problem that can lead to cardiovascular disease, hypertension, diabetes, and other metabolic diseases [1, 2]. Current research has reported that obesity is a chronic inflammatory state, which is closely related with excessive accumulation of free fatty acid (FFA) due to lipolysis of adipocytes [1, 3]. The excessive accumulation of FFA will cause the activation of inflammatory signaling pathways, finally causing cell dysfunction [4]. To date, the specific mechanism that high level of FFA causes inflammation is not very clear.

Kruppel-like factors (KLFs), as a transcription factor family, are composed of 17 members with zinc finger structure, widely involving cell proliferation, differentiation, and embryonic developmental regulation [5]. KLF4, originally separated from the gastrointestinal tract, is one of the transcriptional regulation factors of adipocyte differentiation [6]. In recent years, KLF4 has involved the regulation effect of various chronic inflammatory responses. In vascular endothelial cell, KLF4 combined with the activity subunit P65 of nuclear transcription factor *κ*B (NF-*κ*B), which caused the inhibition of the combination of P65 and vascular cell adhesion molecule 1 (VCAM1) to play an anti-inflammatory role [7]. Recently, it was reported that the overexpression of KLF4 increased the M2 macrophage (anti-inflammation) marker protein expression, while it decreased M1 macrophage (inflammation) marker protein expression. Moreover, KLF4-deficient macrophages exhibited lower ability to perform fatty acid oxidation [8]. In J774a cells, KLF4 overexpression reduced the expression of MCP-1 [8]. In adipocytes, whether KLF4 plays an important role in FFA induced inflammatory response has not been reported yet.

Toll-like receptors (TLRs) play an important role in inflammatory signaling pathways of congenital immune response. Latest literature reported that *TLR9* gene knockout mice with a high-fat diet had more visceral fat accumulation and released inflammatory factors, such as IL-6, MCP-1 and TNF-*α* [9]. In human lung epithelial cell, TLR9 raised KLF4 expression through MYD88/SRC pathway to promote the release of anti-inflammatory factor IL-10 [10]. In adipocytes, whether TLR9/KLF4 plays an anti-inflammatory role in FFA-induced inflammatory response has not been reported.

Therefore, on the basis of constructing high-fat diet-induced obese rat model, studying the human omental adipose tissue, and culturing adipocytes in vitro, our research intended to explore the molecular mechanism of the inhibition effect of TLR9/KLF4 on FFA-induced inflammatory response of adipocytes, which can help to elucidate the molecular mechanism of obesity initiating inflammation.

## 2. Materials and Methods

### 2.1. Source of Tissue Samples

From January to December 2014, 95 individual participants were enrolled with ages between 20 and 90 years old in the People's Hospital of Xinjiang Uygur Autonomous Region for physical examination and evaluation of dyslipidemia. They were divided into two groups: the normal control group (NC, *n* = 50, 18.0 kg/m^2^ ≤ BMI ≤ 23.9 kg/m^2^) and the obese group (OB, *n* = 45, BMI ≥ 28 kg/m^2^). Thirty-two Wistar male rats provided by the Centers for Disease Control in Xinjiang Uygur Autonomous Region were fed in the Center for Experimental Animals of Shihezi University. The rats were divided into two groups: high-fat diet (HFD) group (*n* = 16) and normal diet control group (*n* = 16). This study was approved by the medical ethics committee (no. 2014LL22), and the participants all signed an informed consent.

### 2.2. Cell Lines and Plasmid

The 3T3-L1 cell lines were bought from the Cell Center of Basic Medical Institute of the Chinese Academy of Medical Sciences. pIRES2, pIRES2-KLF4, TLR9 lentiviral vector, and siRNA of TLR9 and KLF4 were bought from Gemma Pharmaceutical Technology.

### 2.3. Real-Time PCR

Real-time PCR was performed using SYBR Premix Ex Taq (Takara) on a 7500 Real-Time PCR System (Applied Biosystems, Foster City, CA). Primer sequences are listed in Table S1, using *GAPDH* or *β-actin* as an internal control.

### 2.4. Cell Culture and Differentiation

The 3T3-L1 cells were cultured in DMEM supplemented with 10% fetal bovine serum and antibiotics. Culturing preadipocyte 3T3-L1 was done to differentiate into mature adipocytes which is identified by oil red O.

### 2.5. Transfection Assays

All transient transfection and adenoviral overexpression procedures were performed in 3T3-L1 cell line. For overexpression, 3T3-L1 cells were infected with the empty control virus or the lentivirus carrying the mouse TLR9 gene and the pIRES2-ZsGreen1 or pIRES2-KLF4-ZsGreen1. Transient transfection assays were done with lipo2000 transfection reagent (Invitrogen, USA). For downexpression, 3T3-L1 cells were infected with small interference RNA.

### 2.6. Western Blot

The total protein was analyzed by sodium dodecyl sulphate-polyacrylamide gel electrophoresis (SDS-PAGE). Membranes were blocked and incubated at 4°C overnight with antibodies. Then, secondary antibody was incubated for 2 h at room temperature. Proteins were visualized using the enhanced chemiluminescence (ECL) detection system (FluorChem HD2, USA).

### 2.7. Participant Consent and Ethics Statement

All participants provided informed and voluntary consent prior to enrolment in the study. This consent included understanding that clinical information and biological samples would be used for research. The consent form and ethical approval were provided by the Medical Ethics Committee at the First Affiliated Hospital, Shihezi University School of Medicine (reference number 2014LL22).

### 2.8. Statistical Analysis

SPSS (version 13.0, SPSS Inc., Chicago, IL, USA) was used for data analysis. Student's *t*-test and rank sum test were used to compare the groups. Correlation analysis was performed using the Pearson method, and a *P* value < 0.05 was defined as statistically significant.

## 3. Results

### 3.1. The mRNA and Protein Expression Levels of TLR9/KLF4 and Inflammation-Related Factors in the Omental Adipose Tissue of Individual Participants

**Figure 1:**
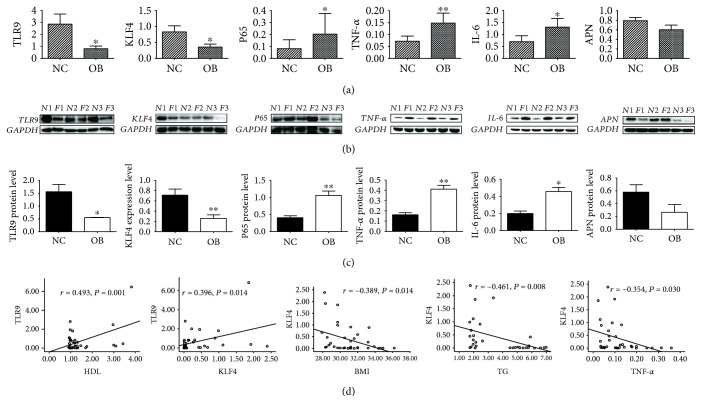
The mRNA and protein expression levels of TLR9, KLF4, and key factors of omental adipose tissue. (a) mRNA expression level; (b) protein expression level; (c) gray scale scanning result; (d) the correlation of TLR9/KLF4 and blood lipid level/key factors of inflammatory signaling pathways in the omental adipose tissue of the OB group. *t*-test, ^∗^ *P* < 0.05, ^∗∗^ *P* < 0.01; the difference was statistically significant; Pearson correlation analysis, *P* < 0.05; the difference was statistically significant.

The clinical characteristics of the individual participants in the NC and OB groups were showed in Tables S2 and S3. In the OB group, the mRNA and protein expression levels of TLR9 and KLF4 were significantly lower than those in the NC group (*P* < 0.05), and APN was lower than the NC group. The mRNA and protein expression levels of subunit p65 of NF-*κ*B, TNF-*α*, and IL-6 in the OB group were significantly higher than those in the NC group (*P* < 0.05) (Figures 1(a)–1(c)). In the OB group, the mRNA expression level of TLR9 was significantly and positively correlated with HDL and KLF4 (*P* < 0.05); the mRNA expression level of KLF4 was significantly and negatively correlated with BMI, TG, and TNF-*α* (*P* < 0.05) (Figure 1(d)).

### 3.2. The mRNA Expression Level of Inflammatory-Related Factors and the Correlation of FFA, TLR9/KLF4, and Inflammatory-Related Genes in the Omental Adipose Tissue of Rats

**Figure 2:**
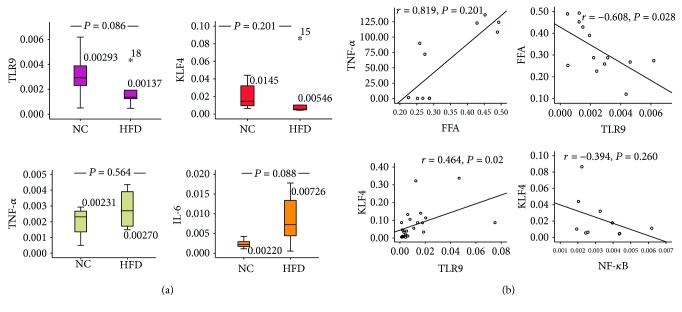
The mRNA expression level and correlation of inflammation-related factors and the correlation of FFA, TLR9/KLF4, and inflammation-related genes in the omental adipose tissue. (a) The mRNA expression levels of TLR9/KLF4 and downstream inflammatory factors; (b) the correlation of TLR9/KLF4 and key genes. Rank sum test, Pearson correlation analysis; *P* < 0.05 was considered statistically significant.

Construction of the obesity animal model was shown in Figure S1 and Table S4. On the basis of obesity model, we investigated the mRNA expression levels of TLR9/KLF4 and downstream inflammatory-related factors. The mRNA expression levels of TLR9 and KLF4 in the HFD group were lower than those in the NC group, whereas the mRNA expression levels of TNF-*α* and IL-6 in the HFD group were higher than those in the NC group (Figure 2(a)).

The FFA level was significantly and negatively correlated with TLR9 mRNA expression level (*P* < 0.05) and significantly and positively correlated with TNF-*α* (*P* < 0.05); KLF4 was significantly and positively correlated with TLR9 (*P* < 0.05) and was negatively correlated to the subunit P65 of NF-*κ*B (Figure 2(b)).

### 3.3. Culture and Differentiation of 3T3-L1 Adipocytes

**Figure 3:**
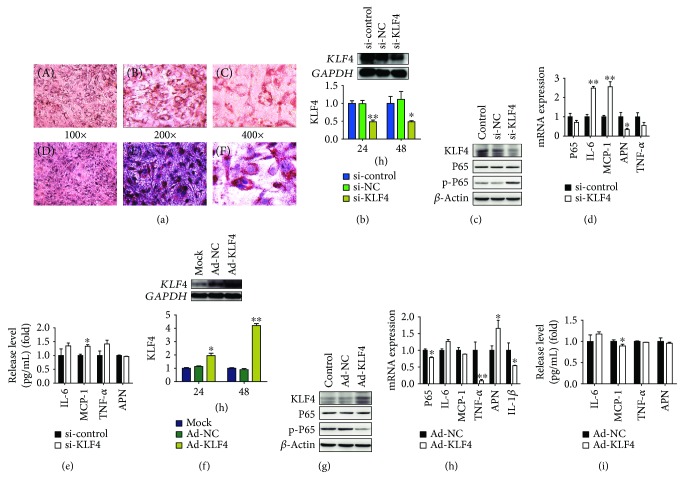
The regulatory effect of KLF4 on downstream inflammatory factors. (a) The differentiation of 3T3-L1 adipocytes; (b) the mRNA and protein expression level of KLF4 after KLF4 interference for 24 h and 48 h; (c) detecting the protein expression level of p-P65 after downregulation of KLF4; (d) detecting the mRNA expression levels of P65, IL-6, MCP-1, TNF-*α*, and APN after downregulation of KLF4; (e) release levels of IL-6, MCP-1, TNF-*α*, and APN after downregulation of KLF4; (f) mRNA and protein expression levels of KLF4 after pIRES2-KLF4 plasmid was transfected into mature adipocytes after 24 h and 48 h; (g) detecting the protein expression level of p-P65 after upregulation of KLF4; (h) mRNA expression levels of P65, IL-6, MCP-1, TNF-*α*, APN, and IL-1*β*; (i) detecting the release levels of IL-6, MCP-1, TNF-*α*, and APN after upregulation of KLF4. *t-*test, ^∗∗^ *P* < 0.01, ^∗^ *P* < 0.05; the difference has a statistical significance. The effects on KLF4 expression after upregulation of TLR9.

The 3T3-L1 cell is fusiform and lipid droplet does not exist. On differentiation by the 8th day, there were more than 90% mature adipocytes, gathering more lipid droplet. The oil red O staining results showed that the lipid droplet was dyed red. Hematoxylin stain results showed that the nuclear was dyed purple (Figure 3(a)).

### 3.4. The Effects on Downstream Inflammatory Factors after Downregulation of KLF4

After KLF4 interference for 24 h and 48 h, the KLF4 mRNA expression level was significantly lower than the si-control and si-NC groups (*P* < 0.05). After KLF4 interference for 24 h, the protein expression level of KLF4 was also lower than the si-control and si-NC groups (Figure 3(b)). Next, we examined the mRNA expression level and release level of inflammation-related factors after KLF4 interference for 24 h. The results showed that the protein expression level of p-P65 was significantly lower than that of the Ad-NC group (Figure 3(c)), and the mRNA expression levels of IL-6 and MCP-1 were significantly increased compared with those of the si-control group (*P* < 0.01), while the mRNA expression level of anti-inflammatory factor APN was significantly decreased compared with that of the si-control group (*P* < 0.05) (Figure 3(d)). After KLF4 interference, the release level of MCP-1 was also significantly increased (*P* < 0.01) (Figure 3(e)).

### 3.5. The Effects on Downstream Inflammatory Factors after Upregulation of KLF4

After pIRES2-KLF4 plasmid was transfected into mature adipocytes after 24 h and 48 h, the mRNA expression levels of KLF4 were both significantly increased (*P* < 0.05), and in the 48 h transfection, the KLF4 mRNA expression level was significantly higher than that in the 24 h transfection group (*P* < 0.05). Moreover, the KLF4 protein expression level was also significantly increased in the 48 h transfection group (Figure 3(f)). Therefore, we investigated the inflammation-related factor expression and release level in the group of 48 h transfection of KLF4 plasmid. The results showed that the protein expression level of p-P65 was significantly lower than that of the Ad-NC group (Figure 3(g)), and the mRNA expression levels of inflammatory-related factors, such as P65, TNF-*α*, and IL-1*β*, were significantly lower than those of the Ad-NC group (*P* < 0.05), while the mRNA expression level of anti-inflammatory factor APN was significantly higher than that of the Ad-NC group (*P* < 0.05) (Figure 3(h)). After the upregulation of KLF4, the release level of MCP-1 was significantly decreased (*P* < 0.05) (Figure 3(i)).

### 3.6. The Effects on KLF4 Expression after Upregulation of TLR9

**Figure 4:**
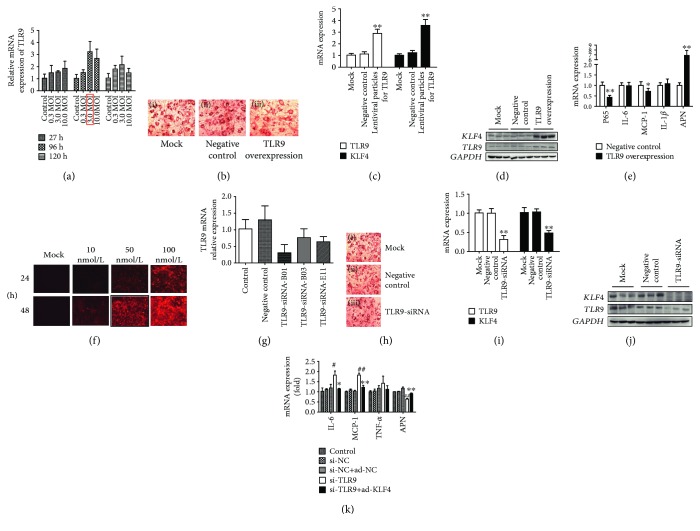
Detection of TLR9 regulation on KLF4 and key factors. (a) TLR9 mRNA expression level in MOI = 0.3, 3, and 10 and time = 72, 96, and 120 h, respectively; (b) oil red O staining results of the mock, negative control, and virus infection groups; (c, d) upregulated TLR9, detecting theTLR9 and KLF4 mRNA and protein expression levels; (e) upregulated TLR9, detecting the mRNA expression level of P65, IL-6, MCP-1, IL-1*β*, and APN; (f) the fluorescence in concentration = 10, 50, and 100 nmol/L and time = 24 h and 48 h; (g) the interference efficiency of three different TLR9-siRNA; (h) oil red O staining results of the mock, negative control, and TLR9-siRNA groups; (i, j) downregulated TLR9, detecting the mRNA and protein expression levels of TLR9 and KLF4. *t*-test, ^∗∗^ *P* < 0.01, ^∗^ *P* < 0.05; the difference has a statistical significance; (k) overexpressing KLF4 while knocking down TLR9, detecting the mRNA expression level of IL-6, MCP-1, TNF-*α*, and APN. ANOVA test, ^##^ *P* < 0.01, ^#^ *P* < 0.01 compared with si-NC; ^∗∗^ *P* < 0.01, ^∗^ *P* < 0.05 compared with si-TLR9.

First, the MO1 numbers 0.3, 3, and 10, respectively, were infected into mature adipocytes for 72 h, 96 h, and 120 h. The results showed that the mRNA expression level of TLR9 was highest when the MO1 was 3 and infected for 96 h (Figure 4(a)). Therefore, we examined the TLR9 regulation on KLF4 under this condition. The results showed that the number of adipocytes has no significant difference compared with the mock and negative control group after TLR9 overexpression. Moreover, TLR9 mRNA and protein expression levels were both significantly higher than the mock and negative control group after TLR9 overexpression (*P* < 0.01). After upregulation of TLR9, the mRNA and protein levels of KLF4 were significantly higher than those of the mock and negative control groups (*P* < 0.01) (Figures 4(c) and 4(d)). Compared with the mock and negative control groups, upregulated TLR9 can significantly decrease the mRNA expression levels of P65 and MCP-1 (*P* < 0.05), while significantly increase the expression level of APN (*P* < 0.01) (Figure 4(e)).

### 3.7. The Effects on KLF4 Expression after Downregulation of TLR9

The negative interference fragment with red fluorescence was transfected into mature adipocytes, and the concentration gradients were 10, 50, and 100 nmol/L. Then, we observed the transfection efficiency in 24 h and 48 h. The results showed that with the concentration increasing, the transfection efficiency was also increased, and the fluorescence intensity in the 48 h transfection group was significantly higher than that in the 24 h group. When the concentration of interference fragment was 50 nmol/L and the interference time was 48 h, fluorescence intensity had reached 80-90% (Figure 4(f)). Next, we screened the best interference fragment from three TLR9-siRNA interference fragments, and the results showed that TLR9-siRNA-B01 had the highest efficiency (Figure 4(g)). The number of adipocyte has no significant difference compared with the mock and negative control groups after TLR9-siRNA-B01 fragment transfection (Figure 4(h)). Moreover, the mRNA and protein expression levels of TLR9 were significantly lower than those of the mock and negative control groups after TLR9-siRNA-B01 fragment transfection (*P* < 0.05). The mRNA and protein expression levels of KLF4 were significantly lower than those of the mock and negative control groups after downregulation of TLR9 (Figures 4(i) and 4(j)). Downregulated TLR9 can significantly increase the mRNA expression levels of subunit P65 of NF-*κ*B, IL-6, and MCP-1 (*P* < 0.05), while the APN is significantly lower (*P* < 0.01). Overexpressing KLF4 while knocking down TLR9, the mRNA expression levels of IL-6 and MCP-1 were significantly descended (*P* < 0.05), while the mRNA expression level of APN was significantly increased (*P* < 0.01) (Figure 4(k)).

### 3.8. Under Different Concentrations of PA Stimulation, the Expression Levels of TLR9/KLF4 and Inflammatory Cytokines Were Detected

**Figure 5:**
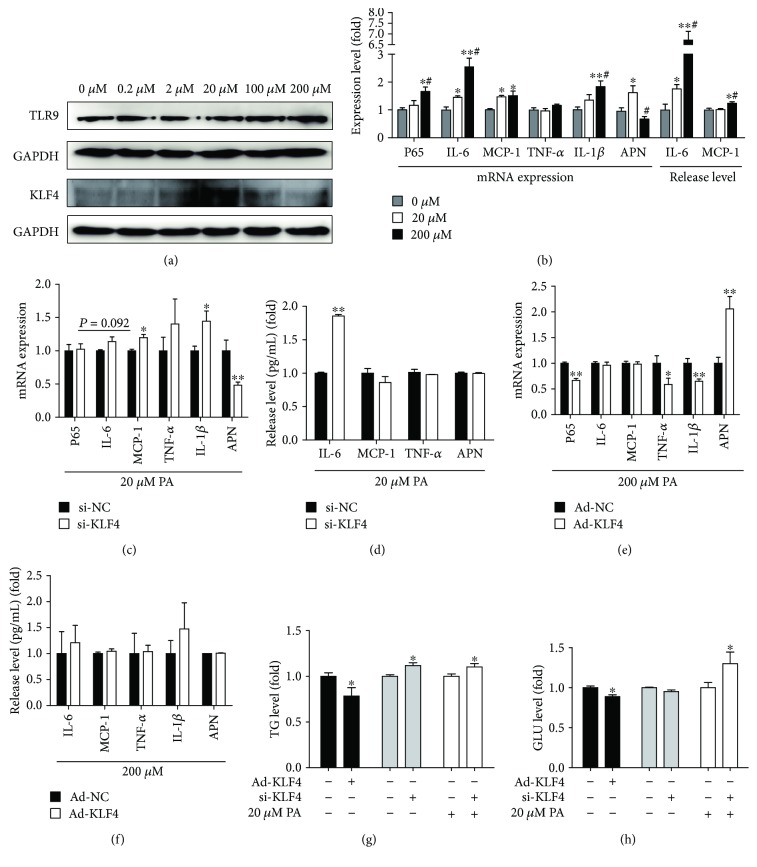
High FFA promotes the expression level of inflammation factors by downregulating KLF4. (a) Different concentrations of PA were used to stimulate cells to detect the protein expression levels of TLR9/KLF4; (b) the mRNA expression levels of P65, IL-6, MCP-1, TNF-*α*, APN, and IL-1*β* and the release expression levels of IL-6 and MCP-1 under the 20 *μ*M and 200 *μ*M PA stimulation; (c) detecting the mRNA expression levels of P65, IL-6, MCP-1, TNF-*α*, APN, and IL-1*β* after 20 *μ*M PA stimulation and si-KLF4; (d) detecting the release expression levels of IL-6, MCP-1, TNF-*α*, and APN after 20 *μ*M PA stimulation and si-KLF4; (e) detecting the mRNA expression levels of P65, IL-6, MCP-1, TNF-*α*, APN, and IL-1*β* after 200 *μ*M PA stimulation and KLF4 transfection; (f) detecting the release expression levels of IL-6, MCP-1, TNF-*α*, and APN after 200 *μ*M PA stimulation and KLF4 transfection; (g, h) the levels of TG and GLU after 20 *μ*M PA stimulation and si-KLF4. *t*-test, ^∗∗^ *P* < 0.01, ^∗^ *P* < 0.05, ^#^ *P* < 0.05; the difference has a statistical significance.

Selecting different concentrations of saturated fatty acid PA (0, 0.2, 2, 20, 100, and 200 *μ*M) stimulates adipocytes for 48 h to detect the mRNA and protein expression levels of TLR9/KLF4. We found that with the increase of PA concentration, the protein expression level of TLR9 had no statistical significance, while the level of KLF4 was increased at first and then decreased. When the PA concentration is 20 *μ*M, the KLF4 expression level was the highest, and the difference was statistically significant compared with the NC group (*P* < 0.05). Under the 100 *μ*M and 200 *μ*M PA stimulation, the mRNA and protein expression levels of KLF4 were significantly decreased compared with those of the 20 *μ*M PA stimulation (*P* < 0.05) (Figure 5(a)). In the 20 *μ*M PA stimulation group, the mRNA expression levels of IL-6, MCP-1, and APN and the release level of IL-6 were significantly higher than those in the 0 *μ*M PA stimulation group. In the 200 *μ*M PA stimulation group, the mRNA expression levels of P65, IL-6, MCP-1, and IL-1*β* and the release levels of IL-6 and MCP-1 were significantly higher than those in the 0 *μ*M PA stimulation group. In the 200 *μ*M PA stimulation group, the mRNA expression level of IL-6 and the release levels of IL-6 and MCP-1 were significantly higher than those in the 20 *μ*M PA stimulation group, while the mRNA expression level of APN was significantly lower than that of the 20 *μ*M PA stimulation group, and the above differences were all statistically significant (*P* < 0.05) (Figure 5(b)).

### 3.9. Detecting the Expression of Downstream Inflammatory Factors under the 20 *μ*M PA Stimulation and Downregulated KLF4

Under the 20 *μ*M PA stimulation with downregulated KLF4, the mRNA and protein expression levels of KLF4 were significantly decreased (*P* < 0.01) (Figure S2). Next, we investigated the expression of downstream inflammatory factors. We found that under the 20 *μ*M PA stimulation, downregulated KLF4 increased the mRNA expression levels of MCP-1 and IL-1*β* and the release level of IL-6 (*P* < 0.05), while it inhibited the mRNA expression level of the APN (*P* < 0.01) (Figures 5(c) and 5(d)).

### 3.10. Detecting the Expression of Downstream Inflammatory Factors under the 200 *μ*M PA Stimulation and Upregulated KLF4

Under the 200 *μ*M PA stimulation with upregulated KLF4, the mRNA and protein expression levels of KLF4 were significantly increased (*P* < 0.01) (Figure S3). Next, we examined the expression of downstream inflammatory factors. We found that under the 200 *μ*M PA stimulation, upregulated KLF4 could significantly suppress the expression levels of subunit P65 of NF-*κ*B, TNF-*α*, and IL-1*β*, while it increased the mRNA expression level of APN (*P* < 0.05) (Figures 5(e) and 5(f)).

### 3.11. Detecting the KLF4 Effects on Glycolipid Metabolism Ability

In culture medium, upregulated KLF4 could significantly suppress the level of TG and GLU (*P* < 0.05), and downregulated KLF4 could significantly increase the level of TG (*P* < 0.05). Under the 20 *μ*M PA stimulation, downregulated KLF4 could significantly increase the level of TG and GLU (*P* < 0.05) (Figures 5(g) and 5(h)).

## 4. Discussion

Obesity is a state of chronic inflammation and is closely related to insulin resistance, type 2 diabetes, and other metabolic diseases [11]. The accumulation of FFA plays an important role in the inflammatory response. Therefore, investigating the specific molecular mechanism of FFA-induced inflammatory response of the adipose tissue will provide theoretical basis for the treatment of obesity and metabolic disorders. Thus, on the basis of constructing high-fat diet-induced obese rat model, the human omental adipose tissue, and culturing adipocytes in vitro, our research intended to explore the molecular mechanism of the inhibition effect of TLR9/KLF4 on FFA-induced inflammatory response of adipocytes.

### 4.1. TLR9 Can Promote KLF4 Expression to Exert Anti-Inflammatory Effect

Existing literature reported that KLF4, as a transcription factor, plays an anti-inflammatory role in the different cell types, such as vascular endothelial cell, lung epithelial cell, and macrophage [5–8]. Whether KLF4 plays an anti-inflammatory role in adipocytes has not been reported yet. Our results showed that downregulated KLF4 promoted the expression of inflammatory cytokines IL-6 and MCP-1. Upregulated KLF4 inhibited the expression of inflammatory cytokines IL-1*β* and MCP-1. The above results suggested that KLF4 played an anti-inflammatory role in adipocytes. Existing literature showed that TLR9 could inhibit inflammatory reaction in adipose tissues, colitis tissues, intestinal tissues, and human lung epithelial cells [12]. Our results showed that downregulated TLR9 significantly decreased the mRNA and protein expression levels of KLF4 and anti-inflammatory factor APN, while it significantly promoted the inflammatory factor expression. These results suggest that TLR9 plays an anti-inflammatory role by increasing KLF4 expression.

### 4.2. High Concentration of PA Inhibits KLF4, While It Has No Effects on TLR9

Due to the important role of FFA in obesity-induced inflammation, we then investigated the FFA effects on TLR9/KLF4. In individual participants, we found that the levels of TG and LDL were significantly higher in the OB group, the mRNA and protein expression levels of KLF4 were significantly lower in the OB group compared with the NC group, and KLF4 was significantly and negatively correlated with TG. The neutral fat including TG can be decomposed into FFA that is a key factor of inflammation. The above results suggested that high concentration of FFA might inhibit the expression level of KLF4. In the NC group, the TG level was lower, while the expression level of KLF4 was higher, which suggested that low concentration of FFA might promote the expression level of KLF4. Recent research also put forward that KLF4 knockout could inhibit the lipid intake and fatty acid oxidation ability in macrophagocyte [8]. Moreover, the elongation of long-chain fatty acid family member 6 (Elovl6) can suppress the KLF4 expression [13]. Thus, we investigated the expression level of KLF4 and inflammatory factors under the different concentrations of PA stimulated in adipocytes. The results showed that under the low concentration of PA stimulation, the high expression level of KLF4 could improve the glucolipid metabolism ability. However, under high concentrations of PA stimulation, the lower expression level of KLF4 could promote the expression of inflammatory factors IL-6, IL-1*β*, and MCP-1. The above results suggest that high PA concentration promotes the expression of inflammatory factor by inhibiting KLF4.

In the adipose tissue of the OB group of individual participants, TLR9 mRNA and protein expression levels were significantly lower than those of the NC group, and the mRNA expression level of TLR9 was significantly and positively correlated with HDL and KLF4. Meanwhile, in the adipose tissue of the HFD group of rats, the mRNA expression level of TLR9 was lower than that of the NC group and was significantly and negatively correlated with FFA while positively correlated with KLF4. The above results suggest that abnormal blood lipid level was significantly correlated with TLR9 and KLF4 expression in the adipose tissue. In adipocyte culture, we found that TLR9 positively promoted KLF4 expression, and while under different concentrations of PA, the TLR9 expression level has no significant difference. The above results suggest that high PA concentration that inhibits KLF4 expression was not via TLR9. So, how does the PA inhibit KLF4? DNA methylation is an important means of regulation of eukaryotic gene expression and a bridge that connects environment change and cellular response. Existing literature reported that high-fat diet can lead to many metabolism-related gene methylation status [14, 15]. Whether PA inhibits KLF4 expression due to the gene methylation status changing should be studied further.

In vivo, we found that TLR9 was significantly negatively correlated with FFA, while PA did not regulate TLR9 in vitro, which is inconsistent. The reason we suspect is the different kinds of FFA. There are 37 kinds of FFA, and TLR9 expression may be correlated with other fatty acid but not PA, such as oleic acid and arachidonic acid. The specific mechanism needs to be further studied.

### 4.3. KLF4 Upregulation of the Expression Level of APN

APN, specifically secreted by adipocytes, is an anti-inflammatory molecule that can suppress immune response and atherosclerosis and is negatively correlated with varieties of metabolic diseases caused by obesity [16]. Our results showed that compared with the NC group, the expression level of APN was lower in the OB group. In adipocytes, high PA concentration inhibited APN expression. Upregulated KLF4 could significantly increase the level of APN, and downregulated KLF4 could significantly decrease the level of APN. Moreover, under the high concentration of PA stimulation, KLF4 overexpression could significantly increase the APN expression. Under the low concentration of PA stimulation, KLF4 downexpression could significantly reduce APN expression. The above results indicated that high levels of FFA inhibited the expression level of APN through downregulating KLF4, eventually leading to the inflammatory reaction. KLF4 as a transcriptional regulation factor, and whether KLF4 directly regulated *APN* gene expression needs further research.

In summary, our research has explored the molecular mechanism of the inhibition effect of TLR9/KLF4 on FFA-induced inflammatory response of adipocytes, which can help to elucidate the molecular mechanism of obesity initiating inflammation and to provide a new experimental basis. Although our research found that FFA promoted inflammation by inhibiting TLR9/KLF4 in adipocytes, we did not exclude the role of key factors on the process of inflammation. Therefore, in the future, we still need to further explore their specific molecular mechanism.

## Data Availability

The data of qRT-PCR, Western blot, and ELISA used to support the findings of this study are available from the corresponding author upon request.

## Supplementary Materials

Supplementary Materials Table S1: primer sequences of fragments. Table S2: comparison of subject metrics and biochemical parameters between the NC and the OB group in the individuals. Table S3: comparison of biochemical indexes and inflammatory factor level between the NC and the OB group in the individuals. Table S4: the comparison of blood glucose, lipid, adipocytokines, and inflammatory factor level in the rat model of obesity. Figure S1: construction of obese animal model. The rats were monitored for 10 weeks while being fed normal diet and high-fat diet. (A) Body weight (*n* = 8 rat per group). (B) The appearance of the rat. Scale bar: 1 cm. (C) The visceral fat mass and liver appearance. Scale bar: 1 cm. *t*-test, values are given as the mean ± SD. ^∗^ *P* < 0.05, ^∗∗^ *P* < 0.01 compared with the NC group. Figure S2: the expression levels of KLF4 after 20 *μ*M PA stimulation and si-KLF4. (A) mRNA expression level of KLF4. (B) Protein expression level of KLF4. *t*-test, ^∗∗^ *P* < 0.01, ^∗^ *P* < 0.05; the difference has a statistical significance. Figure S3: the expression levels of KLF4 after 200 *μ*M PA stimulation and KLF4 transfection. (A) mRNA expression level of KLF4. (B) Protein expression level of KLF4. *t*-test, ^∗∗^ *P* < 0.01, ^∗^ *P* < 0.05; the difference has a statistical significance.
