# Supplementary material for: The Effect and Mechanism of TLR9/KLF4 in FFA-Induced Adipocyte Inflammation
Source: Mediators Inflamm. 2018 Dec 18;2018:6313484. doi: 10.1155/2018/6313484 (PMC6312605; doi:10.1155/2018/6313484)
Supplement: Supplementary Materials — Table S1: primer sequences of fragments. Table S2: comparison of subject metrics and biochemical parameters between the NC and the OB group in the individuals. Table S3: comparison of biochemical indexes and inflammatory factor level between the NC and the OB group in the individuals. Table S4: the comparison of blood glucose, lipid, adipocytokines, and inflammatory factor level in the rat model of obesity. Figure S1: construction of obese animal model. The rats were monitored for 10 weeks while being fed normal diet and high-fat diet. (A) Body weight (n = 8 rat per group). (B) The appearance of the rat. Scale bar: 1 cm. (C) The visceral fat mass and liver appearance. Scale bar: 1 cm. t-test, values are given as the mean ± SD. ∗ P < 0.05, ∗∗ P < 0.01 compared with the NC group. Figure S2: the expression levels of KLF4 after 20 μM PA stimulation and si-KLF4. (A) mRNA expression level of KLF4. (B) Protein expression level of KLF4. t-test, ∗∗ P < 0.01, ∗ P < 0.05; the difference has a statistical significance. Figure S3: the expression levels of KLF4 after 200 μM PA stimulation and KLF4 transfection. (A) mRNA expression level of KLF4. (B) Protein expression level of KLF4. t-test, ∗∗ P < 0.01, ∗ P < 0.05; the difference has a statistical significance. [file 6313484.f1.doc]

**Primer sequences of fragments.** PCR amplification primers name, sequences and fragments length of all genes are shown in Table S1.

**Table S1 Primer sequences of fragments**

| **Species** | **Primers name** | **Sequences (5’→3’)** | **Fragments length** |
| --- | --- | --- | --- |
| **Rat** | *Rat-TLR9-F* | TCCATCATGGTTCTCTGTAGCA | 149bp |
|  | *Rat-TLR9-R* | AGCCAGTTGCAGTCTACCAG |  |
|  | *Rat-KLF4-F* | ACCCCTCTCTCTTCTTCGGA | 192bp |
|  | *Rat-KLF4-R* | GTTCCTCTCGCCAACGGTTA |  |
|  | *Rat-IL-6-F* | CTCTCCGCAAGAGACTTCCAG | 115bp |
|  | *Rat-IL-6-F* | TGTGGGTGGTATCCTCTGTGA |  |
|  | *Rat-TNF-α-F* | TACTCCTCAGAGCCCCCAAT | 120bp |
|  | *Rat-TNF-α-R* | TCAGCGTCTCGTGTGTTTCT |  |
|  | *Rat-β-actin-F* | AGGCCGGCTTCGCGGGCGAC | 244bp |
|  | *Rat-β-actin-R* | TCAGGGGCCACACGCAGCTC |  |
| **Human** | *Human-TLR9–F* | ACCTCCACACTCACCTCACC | 108bp |
|  | *Human-TLR9–R* | GCCTTCGGTAGCATTTATTGA |  |
|  | *Human-KLF4–F* | GGCACTACCGTAAACACACG | 140bp |
|  | *Human-KLF4–R* | CTGGCAGTGTGGGTCATATC |  |
|  | *Human-NF-κB–F* | CTGAGTCCTGCTCCTTCCA | 103bp |
|  | *Human-NF-κB–R* | CTTCGGTGTAGCCCATTTGT |  |
|  | *Human- TNF-α–F* | GTGACAAGCCTGTAGCCCAT | 111bp |
|  | *Human- TNF-α–R* | TATCTCTCAGCTCCACGCCA |  |
|  | *Human- IL-6–F* | TTCGGTCCAGTTGCCTTCT | 120bp |
|  | *Human- IL-6–R* | GGTGAGTGGCTGTCTGTGTG |  |
|  | *Human- MCP-1–F* | GATCTCAGTGCAGAGGCTCG | 155bp |
|  | *Human- MCP-1–R* | TTTGCTTGTCCAGGTGGTCC |  |
|  | *Human- APN–F* | ATGGCCCCTGCACTACTCTA | 104bp |
|  | *Human- APN–R* | CAGGGATGAGTTCGGCACTT |  |
|  | *Human- GAPDH-F* | GGTGGTCTCCTCTGACTTCAA | 211bp |
|  | *Human-GAPDH-R* | TCTTCCTCTTGTGCTCTTGCT |  |
| **Mouse** | *Mouse-TLR9–F* | GAATCCTCCATCTCCCAACAT | 92bp |
|  | *Mouse-TLR9–R* | CCAGAGTCTCAGCCAGCACT |  |
|  | *Mouse-KLF4–F* | CTGAACAGCAGGGACTGTCA | 218bp |
|  | *Mouse-KLF4–R* | GTGTGGGTGGCTGTTCTTTT |  |
|  | *Mouse-TNF-α–F* | CTGTAGCCCACGTCGTAGC | 97bp |
|  | *Mouse-TNF-α–R* | TTGAGATCCATGCCGTTG |  |
|  | *Mouse-IL-6–F* | GCTACCAAACTGGATATAATCAGGA | 78bp |
|  | *Mouse-IL-6–R* | CCAGGTAGCTATGGTACTCCAGAA |  |
|  | *Mouse-APN–F* | AGGTTGGATGGCAGGC | 129bp |
|  | *Mouse-APN–R* | GTCTCACCCTTAGGACCAAGAA |  |
|  | *Mouse-MCP-1–F* | ATTGGGATCATCTTGCTGGT | 108bp |
|  | *Mouse-MCP-1–R* | CCTGCTGTTCACAGTTGCC |  |
|  | *Mouse-GAPDH–F* | AACTTTGGCATTGTGGAAGG | 223bp |
|  | *Mouse-GAPDH–R* | ACACATTGGGGGTAGGAACA |  |
| **Interference fragment sequences** | KLF4-MUS-1796 | CACCCACACUUGUGACUAUTT |  |
|  |  | AUAGUCACAAGUGUGGGUGTT |  |
|  | KLF4-MUS-1331 | GGUCAUCAGUGUUAGCAAATT |  |
|  |  | UUUGCUAACACUGAUGACCTT |  |
|  | KLF4-MUS-901 | CCUCCUGGACCUAGACUUUTT |  |
|  |  | AAAGUCUAGGUCCAGGAGGTT |  |
|  | TLR9-MUS-E11 | CCAACAUCCUGGUUCUAGAUGCUAA |  |
|  |  | UUAGCAUCUAGAACCAGGAUGUUGG |  |
|  | TLR9-MUS-B01 | GCCAGCCCUUUAGCAUGAAGGGUAU |  |
|  |  | AUACCCUUCAUGCUAAAGGGCUGGC |  |
|  | TLR9-MUS-B03 | UGGUGGUGCCUAUACUGCACCAUCU |  |
|  |  | AGAUGGUGCAGUAUAGGCACCACCA |  |

**Comparison of subject metrics and biochemical parameters between NC and OB group in the individuals.** The weight, WC, HC, WHR, and BMI of individuals in the OB group were significantly higher than those in the NC group (*P*<0.05)(Table S2).

**Table S2 Comparison of subject metrics and biochemical parameters**

**between NC and OB group in the individuals**

| **Testing index** | **NC** | **OB** |
| --- | --- | --- |
| **Case number** | 50 | 45 |
| **Age** | 47.42±17.39 | 45.94±10.01 |
| **weight（kg）** | 63.08±7.74 | ***79.96±11.10***** |
| **WC（cm）** | 89.62±15.29 | ***112.42±8.56***** |
| **HC（cm）** | 93.40±8.64 | ***106.23±18.23***** |
| **WHR** | 0.96±0.16 | ***1.07±0.08***** |
| **BMI** | 22.92±2.54 | ***31.56±3.13***** |
| **SBP(mmHg)** | 120.52±22.61 | 129.74±20.95 |
| **DBP(mmHg)** | 80.28±15.2 | 82.82±14.17 |
| **FPG(mmol/L)** | 5.0±0.85 | 5.0±0.72 |

WC: Waist circumference; HC: Hip circumference; WHR: Waist-to-hip ratio; BMI: Body mass index; SBP: Systolic blood pressure; DBP: Diastolic blood pressure; FPG: Fasting plasma glucose. *t* test, Values are given as the *mean ± SD*. * *P*<0.05, ** *P*<0.01 compared with NC group.

**Comparison of biochemical indexes and inflammatory factor level between NC and OB group in the individuals.** The levels of TG, LDL and TNF-α in the OB group were significantly higher than in the NC group(*P*<0.05)(Table S3).

**Table S3 Comparison of biochemical indexes and inflammatory factor level between NC and OB group in the individuals**

| **Indexes** | **TG(mmol/L)** | **TC(mmol/L)** | **LDL(mmol/L)** | **HDL(mmol/L)** | **TNF-α(μg/mL)** | **APN(μg/mL)** |
| --- | --- | --- | --- | --- | --- | --- |
| **NC(n=50)** | 2.60±1.43 | 4.87±1.20 | 2.64±0.80 | 1.22±0.47 | 20.93±15.32 | 7.36±5.37 |
| **OB(n=45)** | ***3.76±1.89***** | 5.08±1.03 | ***3.02±0.77**** | 1.45±0.80 | ***25.58±14.13**** | 5.71±6.15 |

TG: Triglycerides; TC: Cholesterol; HDL: High density lipoproteins; LDL: Low density lipoproteins. t test, Values are given as the mean ± SD. * *P*<0.05, ** *P*<0.01 compared with NC group.

**The comparison of blood glucose, lipid, adipocytokines and inflammatory factor level in the rat model of obesity.** In the 4^th^ week after high-fat diet feeding, the level of FFA, TG, TC, and LDL in the HFD group were significantly higher than in the NC group (*P*<0.05). In the 10^th^ week, the plasma level of Glu, TG, FFA, and TNF-α in the HFD group were significantly higher than that in the NC group (*P*<0.05); and the level of LPT and APN were significantly lower than the NC group (*P*<0.05) (Table S4).

**Table S4 The comparison of blood glucose, lipid, adipocytokines**

**and inflammatory factor level in the rat model of obesity**

| **Biochemical**  **index** | **The forth week** | | **The tenth week** | |
| --- | --- | --- | --- | --- |
|  | **NC(n=8)** | **HFD(n=8)** | **NC(n=8)** | **HFD(n=8)** |
| **Glu(mmol/L)** | 8.52±0.78 | 8.98±0.96 | 7.32±0.76 | ***8.96±0.72^*^*** |
| **TG(mmol/L)** | 0.58±0.03 | ***0.70±0.07**** | 1.87±0.10 | ***3.27±0.79**** |
| **TC(mmol/L)** | 1.72±0.57 | ***2.39±0.35**** | 1.52±0.34 | 1.57±0.60 |
| **LDL(mmol/L)** | 0.52±0.13 | ***0.91±0.16**** | 0.36±0.06 | 0.45±0.03 |
| **HDL(mmol/L)** | 0.79±0.23 | 0.78±0.19 | 0.80±0.22 | 0.87±0.39 |
| **FFA(mmol/L)** | 0.26±0.02 | ***0.29±0.14**** | 0.33±0.11 | ***0.48±0.08***** |
| **TNF-α(pg/mL)** | 71.45±19.44 | 72.96±26.79 | 79.29±23.84 | ***133.28±20.98**** |
| **LPT(ng/mL)** | 0.39±0.32 | 0.47±0.25 | 0.60±0.55 | ***0.38±0.06**** |
| **APN (mg/L)** | 12.6±0.09 | 12.2±0.14 | 15.6±0.10 | ***11.7±0.20**** |

Glu: glucose; TG: Triglycerides; TC: Cholesterol; LDL: Low density lipoproteins; HDL: High density lipoproteins; FFA: free fatty acids. *t* test, * *P*<0.05 compared with NC group

**Construction of obesity animal model.** At the beginning of the study (weeks 0^th^, 2^th^), there was no statistical difference in rat weight between the NC group and the HFD group (*P*>0.05). In the 4^th^, 6^th^, 8^th^, and 10^th^ week after high-fat diet feeding, the weight of the HFD group was significantly higher than that in the NC group (*P*<0.05). In the 10^th^ week, the weight of the HFD group did not appear to increase, but was still higher than the NC group (*P*<0.01) (Figure S1A). Moreover, compared with the weight of NC group (318.50±38.07), the HFD group (403.00±50.38g) was 20% higher; Moreover, the Lee’s index (337.10±4.72) of the HFD group was significantly higher than the NC group (323.42±4.72) (*P*<0.01); in addition, the waist circumference and visceral fat mass was higher in the HFD mice (Figure S1B/C); and, HFD mice had fatty liver (Figure S1C).


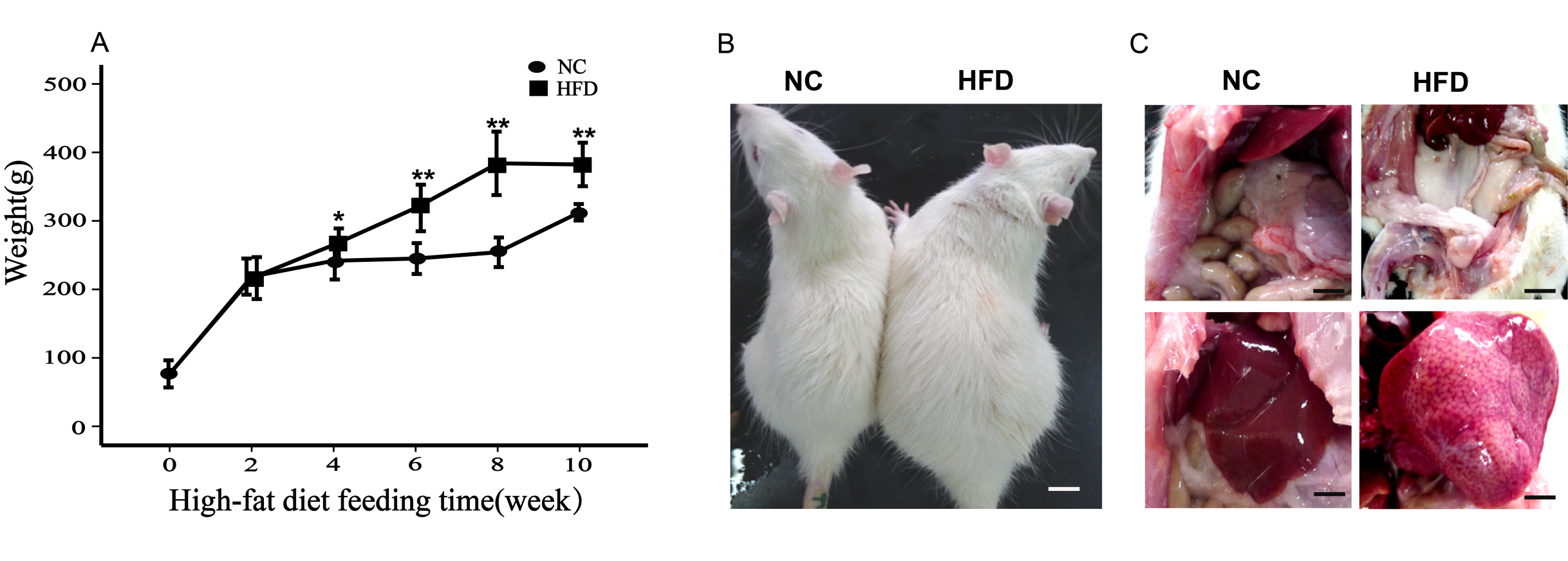


**Figure S1 Construction of obese animal model.** The rats were monitored for 10 weeks while being fed normal diet and high fat diet. (A) Body weight (n=8 rat per group). (B) The appearance of the rat. Scale bar: 1 cm (C) The visceral fat mass and liver appearance. Scale bar: 1 cm. *t* test, Values are given as the *mean* ± *SD*. * *P*<0.05, ** *P*<0.01 compared with NC group.

**The expression levels of KLF4 after 20 μM PA stimulate and si-KLF4.** Under the 20 μM PA stimulate with down-regulated KLF4, the mRNA and protein expression levels of KLF4 were significantly decreased (*P*<0.01) (Figure S2).


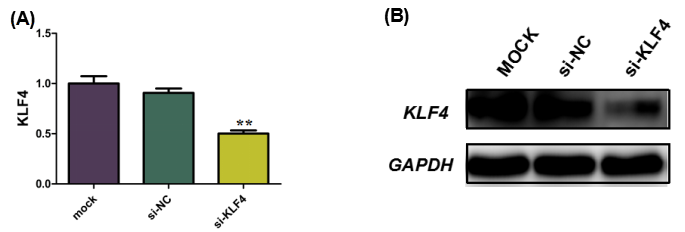


**Figure S2 The expression levels of KLF4 after 20 μM PA stimulate and si-KLF4**

1. mRNA expression level of KLF4 (B) protein expression level of KLF4. *t* test, ***P*<0.01, **P*<0.05 the difference has statistical significance

**The expression levels of KLF4 after 200 μM PA stimulate and transfected KLF4**. Under the 200 μM PA stimulate with up-regulated KLF4, the mRNA and protein expression levels of KLF4 were significantly increased (*P*<0.01) (Figure S3).


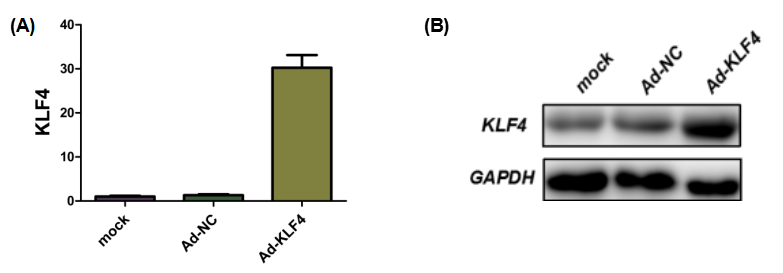


**Figure S3 The expression levels of KLF4 after 200 μM PA stimulate and transfected KLF4**

1. mRNA expression level of KLF4 (B) protein expression level of KLF4

*t* test, ***P*<0.01, **P*<0.05 the difference has statistical significance
